# *In Vitro* Anti-Echinococcal and Metabolic Effects of Metformin Involve Activation of AMP-Activated Protein Kinase in Larval Stages of *Echinococcus granulosus*

**DOI:** 10.1371/journal.pone.0126009

**Published:** 2015-05-12

**Authors:** Julia A. Loos, Andrea C. Cumino

**Affiliations:** 1 Laboratorio de Zoonosis Parasitarias, Departamento de Biología, Facultad de Ciencias Exactas y Naturales, Universidad Nacional de Mar del Plata (UNMdP), Funes 3350, Nivel Cero, (7600), Mar del Plata, Argentina; 2 Consejo Nacional de Investigaciones Científicas y Técnicas (CONICET), Mar del Plata, Argentina; 3 Departamento de Química, Facultad de Ciencias Exactas y Naturales, Universidad Nacional de Mar del Plata (UNMdP), Funes 3350, Nivel 2, (7600), Mar del Plata, Argentina; The University of Hong Kong, HONG KONG

## Abstract

Metformin (Met) is a biguanide anti-hyperglycemic agent, which also exerts antiproliferative effects on cancer cells. This drug inhibits the complex I of the mitochondrial electron transport chain inducing a fall in the cell energy charge and leading 5'-AMP-activated protein kinase (AMPK) activation. AMPK is a highly conserved heterotrimeric complex that coordinates metabolic and growth pathways in order to maintain energy homeostasis and cell survival, mainly under nutritional stress conditions, in a Liver Kinase B1 (LKB1)-dependent manner. This work describes for the first time, the *in vitro* anti-echinococcal effect of Met on *Echinococcus granulosus* larval stages, as well as the molecular characterization of AMPK (Eg-AMPK) in this parasite of clinical importance. The drug exerted a dose-dependent effect on the viability of both larval stages. Based on this, we proceeded with the identification of the genes encoding for the different subunits of Eg-AMPK. We cloned one gene coding for the catalytic subunit (Eg-*ampk*ɑ) and two genes coding for the regulatory subunits (Eg-*ampk*β and Eg-*ampk*γ), all of them constitutively transcribed in *E*. *granulosus* protoscoleces and metacestodes. Their deduced amino acid sequences show all the conserved functional domains, including key amino acids involved in catalytic activity and protein-protein interactions. In protoscoleces, the drug induced the activation of AMPK (Eg-AMPKɑ-P^176^), possibly as a consequence of cellular energy charge depletion evidenced by assays with the fluorescent indicator JC-1. Met also led to carbohydrate starvation, it increased glucogenolysis and homolactic fermentation, and decreased transcription of intermediary metabolism genes. By *in toto* immunolocalization assays, we detected Eg-AMPKɑ-P^176^ expression, both in the nucleus and the cytoplasm of cells as in the larval tegument, the posterior bladder and the calcareous corpuscles of control and Met-treated protoscoleces. Interestingly, expression of Eg-AMPKɑ was observed in the developmental structures during the de-differentiation process from protoscoleces to microcysts. Therefore, the Eg-AMPK expression during the asexual development of *E*. *granulosus*, as well as the *in vitro* synergic therapeutic effects observed in presence of Met plus albendazole sulfoxide (ABZSO), suggest the importance of carrying out chemoprophylactic and clinical efficacy studies combining Met with conventional anti-echinococcal agents to test the potential use of this drug in hydatidosis therapy.

## Introduction

Metformin (1,1dimethylbiguanide, Met) is an oral anti-hyperglycemic agent currently used as the first-choice drug for the treatment of type 2 diabetes, being prescribed to at least 120 million people worldwide. The drug is a synthetic compound derived from the natural product galegine (isoamylene guanidine), extracted from the French lilac or Italian fitch (*Galega officinalis*) as a herbal medicine [1].

The mechanism of action of Met has been studied in the context of diabetes and Met has also been associated with the selective killing of cancer cells [2–6], but it is not yet fully elucidated. Metformin may exert its beneficial metabolic actions through the modulation of multiple components. Its primary function is to decrease hepatic glucose production [7] by reducing the mRNA expression of gluconeogenic genes [2], changing enzyme activities or reducing hepatic uptake of substrates [6]. Besides, Met inhibits the complex I of the mitochondrial electron transport chain, which induces a fall in the cell energy charge [8]. The inhibition of complex I reduces NADH oxidation, lowering the proton-driven synthesis of ATP. Consequently, the ATP:ADP:AMP equilibrium changes towards increased AMP synthesis by adenylate kinase [9, 10]. Metformin also causes the direct inhibition of AMP deaminase, an enzyme that degrades AMP [6, 11]. As a result, AMP levels increase, inducing energy crisis (metabolic stress) and leading to 5'-AMP-activated protein kinase (AMPK) activation [3]. Indeed, Met is recognized as an indirect activator of AMPK by promoting AMP accumulation. On the other hand, AMPK enhances translocation of glucose transporters and directly inhibits raptor, a positive regulator of the target of rapamycin (TOR) in the TOR complex 1 (TORC1) [12]. Also, and independently of AMPK, Met inhibits TORC1 in a Rag-GTPase-dependent manner [4] and both mechanisms exert the anti-proliferative effect of the drug.

AMPK is a master coordinator of metabolic and growth pathways, which induces the catabolic processes that produce ATP and inhibits the anabolic, ATP-consuming processes, in order to restore the cell energy balance. Its primary role lies in the integration of nutrient availability and environmental stress signals associated with the adaptations required to maintain cell homeostasis. This protein is a highly conserved serine/threonine kinase which has orthologs in eukaryotic organisms ranging from yeast (sucrose nonfermenting1-SNF1-), roundworms (AMP-activated kinase-AAK-), and insects to mammals (AMPK) and plants (Snf1-related kinase1-SnRK1-) [13]. AMPK is an obligate heterotrimeric complex comprising a catalytic α-subunit and the regulatory subunits β and γ, which is allosterically activated by AMP and regulated by phosphorylation. The catalytic subunit is highly conserved across multi-species, with a key threonine residue in the activation loop (Thr^210^ in SNF1, Thr^172^ in AMPK and Thr^175^ in SnRK1.1), which requires phosphorylation to confer kinase activity. The β-subunit contains a glycogen-binding domain and a C-terminal domain that mediates the interaction with the α- and γ-subunits of mammalian AMPK [14]. The γ-subunit, with a non-conserved N-terminal region, is characterized by two pairs of cystathionine-beta-synthase repeats that bind adenosine derivates, named Bateman domains. During cell stress, the AMPK complex can undergo a conformational change in which the γ-subunit disrupts its interaction with the catalytic subunit and relieves the inhibition [13]. In mammals, the main upstream kinases are the tumor suppressor Liver Kinase B1 (LKB1), which activates AMPK in response to energy stress, and the Ca^2+/^calmodulin-activated protein kinase kinases (especially CaMKKβ or CaMKK2), which trigger activation in response to increases in cell Ca^2+^ without requiring changes in AMP or ADP levels [15]. Through direct phosphorylation, AMPK exerts control over cellular metabolism and triggers transcriptional reprogramming by recruiting and localizing various transcription factors, such as the forkhead box O (FoxO) proteins [13]. Thus, AMPK, as well as its upstream kinase LKB1 and downstream substrates, which shuttle in and out of the nucleus, can be found both in the nucleus and the cytoplasm [16].

AMPK control increases stress resistance of invertebrate larval stages, such as diapause-like states of many parasitic and free-living nematodes [17]. In the case of parasitic flatworms such as the cestode *Echinococcus granulosus*, whose larval stage causes cystic echinococcosis (CE, hydatidosis) in humans, the functions of AMPK have not yet been elucidated. Human CE is an endemic worldwide zoonosis which involves a complex life cycle with an intermediate host (humans and domestic livestock) and a definitive host (canids). The hydatid cyst or metacestode develops asexually in the intermediate host and produces protoscolex larvae from the inner germinal layer [18]. Most (>90%) CE cysts occur in the liver, lung, or both organs. The parasite shows an alternative development in which protoscoleces released into the circulation after primary cyst surgery or from a ruptured cyst are able to develop into new hydatid cysts, developing secondary CE [18]. At the moment, benzimidazole carbamates (mebendazole, albendazole and its main active metabolite, albendazole sulfoxide-ABZSO-) are the most effective drugs for CE treatment and an alternative to surgery, but the fact that treatment fails in 30–40% of patients encourages the quest for chemotherapeutical alternatives [19]. To develop strategies for CE treatment and control, it is necessary to highlight basic studies on the parasite larval stage and to identify possible new molecular targets. In recent studies, we determined that rapamycin is an effective anti-echinococcal agent and autophagy inducer in *E*. *granulosus* larvae which allowed us to identify TORC1-controlled events in this cestode [20, 21]. Here, we demonstrate that the *E*. *granulosus* larval stage is susceptible to Met *in vitro* and that Met treatment activates Eg-AMPK. We also discuss the results in relation to carbohydrate metabolism, autophagy modulation and developmental processes in the parasite.

## Material and Methods

### Ethics statement

The animal study was performed in strict accordance with National Health Service and Food Quality (SENASA) guidelines, Argentina and with the 2011 revised form of The Guide for the Care and Use of Laboratory Animals published by the U.S. National Institutes of Health. All the experimental protocols were reviewed and approved by the Animal Experimental Committee at the Faculty of Exact and Natural Sciences, Mar del Plata University (permit number: 2555-08-14).

### Experimental animals

Pathogen-free female CF-1 mice (28–35 g), aged 8 weeks, were supplied by the National Health Service and Food Quality-SENASA-. Mice were allowed to acclimatize for one week before starting the experiment. The animals were housed in standard polyethylene cages (five mice per cage) with sawdust (wooden flakes) as nesting material, under controlled laboratory conditions (temperature ±20°C, 12 hour light/12 hour dark with lights off at 8.00 p.m., 55±5% humidity). Water and food pellets were provided *ad libitum* during the study period. Every 3 days, animals were placed into a clean cage with fresh sawdust.

*E*. *granulosus* metacestodes were obtained from the peritoneal cavity of mice injected with 0.5 ml of protoscolex suspension. For each experiment, five experimentally infected mice were killed at 6 months p.i. Animals were anesthetized with ketamine—xylazine (50 mg/kg/mouse—5 mg/kg/mouse) and sacrificed by cervical dislocation. All efforts were made to minimize suffering. Minimum number of animals was used in each experiment.

### *In vitro* culture of protoscoleces, metacestodes and pre-microcyst obtainment

*E*, *granulosus* protoscoleces were removed under aseptic conditions from hydatid cysts of infected cattle presented for routine slaughter at the abattoir (Liminal S. A., official number: 3879) in the province of Buenos Aires, Argentina. Protoscolex *in vitro* culture (*n* = 3,000/9.5-cm^2^ growth area per well), pharmacological treatment and vitality assays were performed as described below. Otherwise, *E*. *granulosus* metacestodes (10–20 cysts for each drug treatment) were obtained from the peritoneal cavities of CF-1 mice after intraperitoneal infection with protoscoleces [20]. Metformin was purchased from Sigma-Aldrich and ABZSO was kindly provided by C. Salomon, National University of Rosario, Argentina. The drugs were added to the medium either separately or in combination. *In vitro* protoscolex and metacestode treatments were assayed with 1, 5 and 10 mM Met, ABZSO alone at 15 μM (equivalent to 4.2 μg ml^-1^), and the combination of 1, 5 and 10 mM Met plus 15 μM ABZSO for until 15 and 7 days, respectively [21]. In both cases, viability was assessed daily until the viability control was lower than 90%. Protoscolex viability assessment was determined by the methylene blue exclusion test [20] and cyst viability measurement was evaluated through an inverted light microscope having as criteria the collapse of the germinal layers and the cell viability with the trypan blue exclusion test from a detached germinal membrane. For scanning electron microscopy (SEM), samples were taken every 24 h and processed as previously described [22]. Each viability experiment was performed using three replicates per treatment condition and repeated three times. For molecular and biochemical assays, protoscoleces and metacestodes were cultured with 10 mM Met or without drug for 48 h and stored at −80°C until experimental use. Three independent experiments were performed for SEM, enzyme activity, RT-qPCR, confocal microscopy, immunohistochemistry and western blot assays.

In order to obtain vesicularized protoscoleces and pre-microcysts, protoscoleces were cultured in medium 199 supplemented with antibiotics (penicillin, streptomycin and gentamicin; 100 μg/ml), glucose (4 mg/ml), insulin (1.2 U ml^-1^) and 15% FBS as we described in detail previously [21]. Development was followed microscopically under an inverted light microscope every day. Different samples were taken during the pre-microcyst development process and were used for immunohistochemistry studies.

### Enzyme activity analysis and glycogen determination

After two rinses with ice-cold 50 mM Tris—HCl, 0.1–0.2 g of protoscoleces or germinal layers of 5–10 cyst were homogenized in medium containing 50 mM Tris—HCl, 6 mM β-mercaptoethanol, 0.3% (v/v) Triton X-100, 1.5 mM EDTA (pH 7.5 at 4°C) and 1 mM PMSF (phenylmethylsulfonyl fluoride). The suspensions were frozen in liquid nitrogen and thawed at 37°C for three cycles. Then, cells were lysed with a homogenizer with Teflon pestle at 0°C in an ice bath. The homogenate was centrifuged at 100,000 ×g for 15 min and then desalted through Sephadex G-50 columns before the enzyme activity assays. Protein concentrations were quantified by Bio-Rad protein assay kit.

Enzyme activities were determined from protoscolex and metacestode protein extracts following the procedure described below. In all cases, the activities were measured at 37°C in a recording Shimadzu model UV—vis spectrophotometer, the volume of the reaction mixture was 1 ml with 50 μl (100 μg protein) of enzyme samples, and measurements were made after 30 min of incubation. Alpha-amylase activity (1,4-α-D-glucan-4-glucanohydrolase; EC 3.2.1.1) was measured using the 2-chloro-p-nitrophenyl-α-D-maltotrioside (CNP-G3, 2.25 mmol/L) substrate in 100 mM MES buffer (pH 6.0) with 6 mM calcium acetate, 50 mM sodium chloride and 10 mM potassium tiocyanate to release 2-chloro-p-nitrophenol (CNP), resulting in 2-chloro-nitrophenyl-α-Dmaltoside (CNP-G2), maltotriose (G3) and glucose [23]. The absorbance was read at 405 nm against appropriate blanks and the enzyme activity calculated using the molar extinction coefficient for CNP. Lactate dehydrogenase was determined by measuring the formation of the reduced form of nicotinamide adenine dinucleotide (NAD) using 50 mM lactate and 50 mM NAD as substrates in 400 mM methylglucamine (MEG, pH 9.4, Wiener Lab). The rate of NADH formation is directly proportional to the LDH catalytic activity and is determined by measuring the absorbance increase at 340 nm.

Glycogen extraction and quantification from protoscoleces was performed by completely hydrolyzing glucose through an overnight digestion with amyloglucosidase and amylase as previously described [24].

### Gene identification, cloning and expression by reverse transcription (RT)-PCR and quantitative (q)PCR

In order to obtain information on the occurrence of *Echinococcus ampk* sequences, the *E*. *multilocularis* genomic database and *E*. *granulosus* assembled genomic contigs (http://www.sanger.ac.uk/Projects/Echinococcus) were searched with BLASTp and tBLASTn programs. Sequences of *Homo sapiens* and *Bombyx mori* were used as queries. We identified, sequenced and deposited in GenBank a single sequence for each putative gene, including *ampk*α, *ampk*β *and ampk*γ annotated as EgrG_000708800, EgrG_000526000 and EgrG_001024900 in the GeneDB database (with their respective orthologs in *E*. *multilocularis*: EmuJ_000708800, EmuJ_000526000 and EmuJ_001024900) and they were all identified in the recently released whole genome sequence of *Echinococcus* spp. [25]. In addition, homologous genes coding for glucose-6-phosphatase (G6P), fructose-1, 6-bisphosphatase (F1,6BP), phosphoenolpyruvate carboxykinase (PEPCK), α-amylase-like glucosidase and LKB1 were also identified, sequenced and annotated. Specific primers were designed for these genes and the cytoplasmic malate dehydrogenase (*mdh*_*c*_) gene (S1 Table).

Total RNA extractions, RT-PCR, cloning and qPCR were performed as previously described [20]. To analyze the levels of gene expression in control and Met-treated parasites, cDNA was generated from 10 μg of total RNA using Superscript II reverse transcriptase (Invitrogen, Argentina) and Pfu (Promega, USA) DNA polymerase. RT-PCR and qPCR assays were carried out under identical reaction conditions: 30 cycle PCRs of 94°C (30 s), 40°C (1 min), and 72°C (1 min) plus a single step at 72°C for 10 min, their products were analyzed and confirmed as it was previously described [21]. To determine the optimal amount of template, serial 3-fold dilutions of cDNA were carried out. Under these conditions, RT-PCR amplification occurs in the linear range. *E*. *granulosus* actin I (*act*I, GenBank accession no. L07773) was used as an internal control. For qPCR, the calculation of the ratio between the *act*I mean Ct-values in the treated and control sample showed no significant change in gene expression between both samples [26], thus providing a useful internal control in this experiment. The levels of mRNA were normalized to the actin expression level and calculated using the 2^(-ΔΔCT)^ method. Melting curves generated ensure the correct amplification of all genes tested in this work when using the designed primers (S1 Table). PCR amplification efficiency values were near to 96% and the correlation coefficients (r2) between the logarithm of the cDNA starting quantity and the Ct were, at least, 0.95 for all genes.

### Sequence analysis

Ortholog selection was based on reciprocal best BLAST hits and the presence of the characteristic domains in each deduced amino acid sequence. Sequence alignments were generated with the CLUSTALX software program. Nuclear localization signal was predicted with cNLS Mapper Prediction (http://nls-mapper.iab.keio.ac.jp/cgi-bin/NLS_Mapper_form.cgi).

### Studies of Mitochondrial Membrane Potential (ΔΨm)

Control and Met-treated protoscoleces in different times (6-12-24-36 and 48 h) were incubated with 10 mg/mL JC-1 dye for 30 min at room temperature. After incubation, parasites were washed with 20 mM HEPES buffer, pH 7.2, and images were taken using a confocal microscope (Nikon Eclipse C1 Plus). The intensities of green (excitation/emission wavelength = 485/538 nm) and red (excitation/emission wavelength = 485/590 nm) fluorescence were analyzed for 20 individual protoscoleces from control and treated-samples. Images were analyzed using Image J software (NIH). The ratio of red to green fluorescence of JC-1 images was calculated using NIH Image J software (http://rsb.info.nih.gov/ij/).

### Western blot analysis and immunohistochemistry

Polypeptides were separated by SDS—PAGE on 10% polyacrylamide gels and electroblotted onto a nitrocellulose membrane (HyBond C; Amersham, Argentina) as previously described [20]. The membranes were incubated with primary monoclonal antibodies directed against phosphorylated and total human AMPKα [Phospho-AMPKα -Thr172- (40H9) Rabbit mAb and AMPKα (D63G4) Rabbit mAb, Cell Signalling cat no. 2535 and 5832, respectively, USA, 1:1000 dilution] or with primary monoclonal antibody against human actin (JLA-20, Developmental Studies Hybridoma Bank-DSHB, USA, 1:2000 dilution) as a control for protein loading. The anti-AMPKα antibody used in these assays is directed against an epitope which showed 90–97% amino acid identity with the possible ortholog of *E*. *granulosus*. Then, the membranes were incubated with anti-rabbit immunoglobulin (Ig) peroxidase-linked, species-specific whole antibody (GE Healthcare, cat no. NA934V). ECL reagents were used to detect the signals according to the manufacturer’s instructions (GE Healthcare, cat no. RPN2106V1). Chemiluminescence was detected on film and quantified using Image J. To correct any possible unequal loading, each band's density was normalized to its actin density or total target protein. Eg-MDH immunodetection was performed as previously reported [24]. Blots were quantified using an imaging analyzer (Fotodyne model express zoom lens system) and its dedicated software (TotalLab image analysis software) and normalized against actin density.

In parallel, for *in toto* immunohistochemistry, pre-microcysts and control and Met-treated protoscoleces were processed as previously described [21]. Negative controls consisted of omission of primary antibody.

### Statistics

The mRNA expression in protoscoleces and metacestodes was analyzed with the Wilcoxon signed rank nonparametric test. Data within experiments were compared; significance was determined using the student’s *t* test and *P <* 0.05 was considered statistically significant. All data are shown as the arithmetic mean ± SEM.

## Results

### Pharmacological sensitivity of *E. granulosus* protoscoleces and metacestodes to metformin and its combination with albendazole sulfoxide

**Fig 1:**
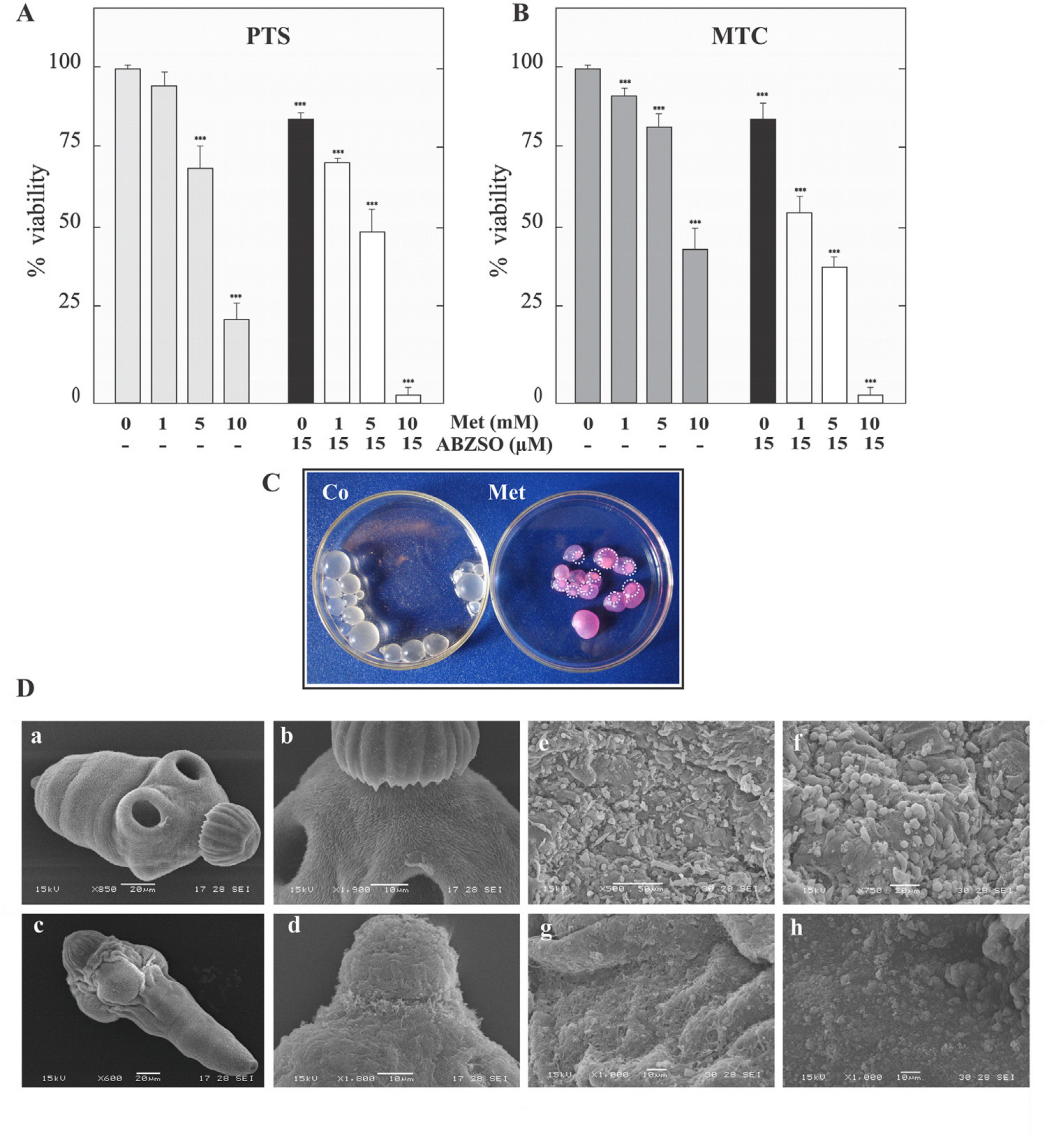
Effect of metformin and its combination with albendazole sulfoxide on viability and ultrastructural characteristics of protoscoleces and metacestodes of *E*. *granulosus*. Viability of protoscoleces (A, PTS) and metacestodes (B, MTC) incubated for 10 and 4 days, respectively with 1, 5 and 10 mM of metformin alone (Met, gray bars), 15 μM albendazole sulfoxide (ABZSO, black bars) alone and 1, 5 and 10 mM Met plus 15 μM ABZSO in combination (open bars). Data are the mean ± S.D. of three independent experiments. ***Statistically significant difference (*P* < 0.05) compared with control. (C) Macroscopical damage of metacestodes treated with 10 mM Met for 4 days. Control metacestodes (Co) without morphological changes and treated metacestodes showing increased permeability (culture medium inside cysts) and collapsed germinal layer (circles). (D) Scanning electron microscopy of protoscoleces (a-d) and metacestodes (e-h) incubated with 10 mM of Met for 4 days. Control protoscolex with normal sucker and microtriches (a,b); treated protoscolex with soma region contracted and scolex region showing loss of hooks and shedding of microtriches (c,d); control murine cyst with an intact germinal layer (e,f); treated cyst with altered germinal layer (g,h). Bars indicate: 10 μm in (b, d and g-h), 20 μm in (a, c and f) and 50 μm (e).

To investigate the *in vitro* effect of Met on the viability of *E*. *granulosus* larval stages, the death percentage of protoscoleces and metacestodes was analyzed in response to various Met concentrations. As shown in Fig 1A and 1B, 10 and 4 days exposure led to a dose-dependent decrease in the viability of protoscoleces and metacestodes, respectively. At 10 mM Met, 80±5% of protoscoleces were dead and 55±5% of metacestodes had disintegrated germinal layers (Fig 1A–1C). During the same time, at 1 mM Met, protoscoleces revealed no changes in vitality in comparison to the control and only 10±2% of metacestodes were dead. In addition, Met-induced damage was observed by SEM after 4 days of treatment with 10 mM drug. In treated protoscoleces, the scolex region was contracted (Fig 1Dc) and rostellar disorganization, loss of hooks and shedding of microtriches were observed (Fig 1Dd), whereas control cultures exhibited no ultrastructural alterations in parasite tissue during the whole incubation period (Fig 1Da-b). Metformin-treated metacestodes revealed loss of cells in the germinal membrane of cysts (Fig 1Dg-h), whereas control metacestodes exhibited an intact germinal layer comprised of a multitude of different, morphologically intact, cell types (Fig 1De-f).

Furthermore, an increased anti-echinococcal effect was found when a combination of Met plus ABZSO was used (Fig 1A and 1B). In this case, 1mM Met plus 15 μM ABZSO (equivalent to 4.2 μg/ml) increased the protoscolex mortality to 30±2% after 10 days of incubation compared with each drug alone, (it was only 20±2% with ABZSO and it did not change with Met). In the case of metacestodes, the mortality increased to 45±5% with 1mM Met plus 15 μM ABZSO in comparison with 20±5% with ABZSO alone 4 days post-incubation.

### Carbohydrate metabolism modifications induced by metformin in *E. granulosus* larval stages

**Fig 2:**
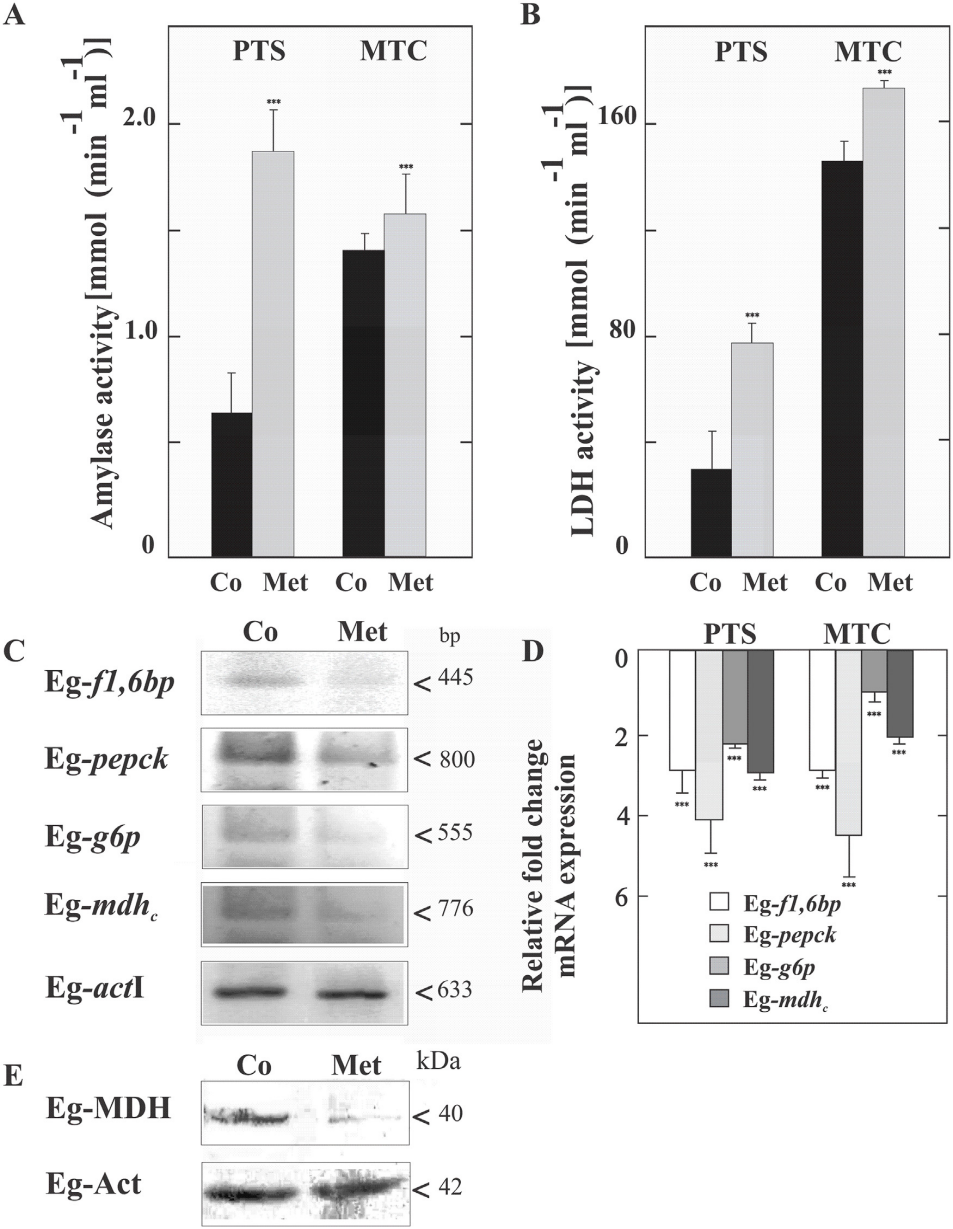
Metabolic and transcriptional changes induced by 10 mM metformin in parasites treated for 48 h. Analysis of Eg-α-amylase (A) and Eg-lactate dehydrogenase (LDH) (B) activities in protoscoleces (PTS) and metacestodes (MTC). (C) Reverse Transcription (RT)-PCR analysis from total RNA of control (Co) or treated (Met) protoscoleces. Amplification of Eg-actin I (*act*I) was used as a loading control. Molecular sizes of amplicons are indicated with arrowheads. Eg-*f1*,*6bp*: fructose-1,6-bisphosphatase, Eg-*pepck*: phosphoenolpyruvate carboxykinase, Eg-*g6p*: glucose-6-phosphatase, Eg-*mdh*_*c*_: cytoplasmic malate dehydrogenase. (D) Quantitative PCR analysis from total RNA of protoscoleces (PTS) and metacestodes (MTC) treated with Met compared to controls. Fold change expression values are plotted. Data are the mean ± S.D. of three independent experiments. ***Statistically significant difference (*P* < 0.05) compared with control. (E) Immunoblot of Eg-MDH revealed with a polyclonal antibody. Total protein extracts from control (Co) and Met-treated protoscoleces (Met) were loaded at 100 μg of total protein/lane. Actin was used as a loading control. Polypeptide size is shown.

To analyze the effect of Met on energy-generating mechanisms in the parasite larvae, we investigated the glycogen levels and the expression and activity of enzymes with a key role in cellular metabolism. A concentration of 10 mM of Met for 48 h revealed low toxicity since the proportion of viable protoscoleces was similar to that of the control (94 ± 3% vs 99%, respectively). However, Met-treated protoscoleces showed a significant decrease in the glycogen level (5.0 ±1.5 mg/g FW-fresh weight-) compared with the control (20± 4 mg/ g FW). In agreement with glycogen degradation, α-amylase and lactate dehydrogenase activities increased upon drug treatment in both larval stages, with a more pronounced percental change in protoscoleces than in metacestodes (Fig 2A and 2B). In addition, in the the *E*. *granulosus* assembled genomic contigs, we identified an ortholog to the *Schistosoma japonicum* α-amylase-like glucosidase gene (CAX75459), which coding sequence was annotated as JN038062 in GenBank (corresponding to Eg-*amyl* gene). The predicted protein sequence (named Eg-α-amylase and annotated as AEJ15816 and EgrG_000494800 in the GenBank and GeneDB database respectively) aligned with at 40% and 35% identity with the *S*. *japonicum* and *Aspergillus oryzae* (0901305A, Taka-Amylase A) orthologs, respectively. *E*. *granulosus*-α-amylase shows three of four catalytic residues invariantly conserved throughout the α-amylase family (pfam00128), corresponding to Asp^229^, Glu^258^ and Asp^323^ and motifs characteristic in six of the seven conserved regions (S1 Fig) [27].

We also analyzed the expression of the Eg-*f1*,*6bp* (annotated as JN038064 in GenBank), Eg-*pepck* (annotated as JN038060), Eg-*g6p* (annotated as JN038058) and Eg-*mdh*_*c*_ genes from protoscoleces and metacestodes. RT-PCR showed a considerable decrease in these transcripts in Met-treated protoscoleces in comparison with the control group (Fig 2C). By qPCR, we found that the transcript levels for Eg-*f1*,*6bp*, Eg-*pepck*, Eg-*g6p* and Eg-*mdh*_*c*_ decreased three-, four-, two- and three-fold in Met-treated protoscoleces and three-, five-, one- and two-fold in Met-treated metacestodes, respectively (Fig 2D). In concordance with gene expression, immunoanalysis of the Eg-MDH polypeptide level showed a considerable decrease in treated protoscoleces respect to the control (c.a. 30% of the control estimated by densitometric analysis normalized to actin, Fig 2E).

### Changes in mitochondrial membrane potential of protoscoleces exposed to metformin

To explore the possible inhibitory effect of Met on the complex 1 of the respiratory chain, we studied the mitochondrial functional status using the membrane potential (ΔΨm) indicator JC-1 in *E*. *granulosus* protoscoleces. JC-1, a positively charged fluorescent compound, can penetrate mitochondria and change their color when the membrane potential increases. In normal mitochondria with high ΔΨm, JC-1 accumulates as aggregates with intense red fluorescence, whereas in damaged mitochondria with low ΔΨm, it remains in the monomeric form, which exhibits only green fluorescence [28].

**Fig 3:**
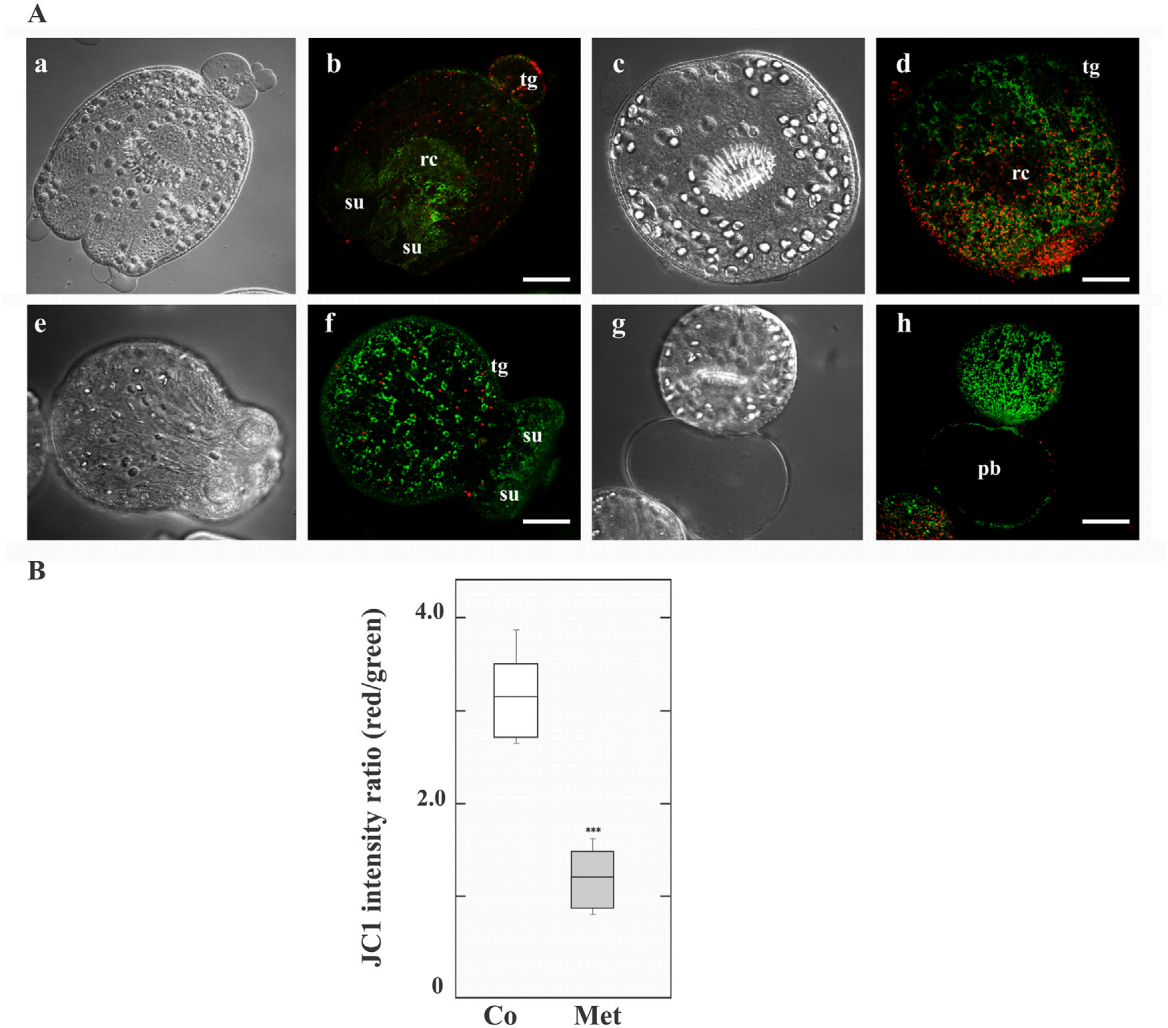
Metformin pharmacological effect on mitochondrial function in protoscoleces. (A) Representative confocal images showing JC-1 fluorescence in protoscoleces incubated under control conditions (a-d) or treated with 10 mM metformin (Met, e-h) for 48 h. tg: tegument; rc: rostellar cone; su: sucker; pb posterior bladder. Bars indicate 50 μm. (B) Boxplot graph showing the values of the red/green JC-1 fluorescence ratios measured in control (Co) and Met-treated protoscoleces by Image J Software. ***Statistically significant difference (*P* < 0.05) compared with control.

Control and Met-treated protoscoleces were examined by confocal microscopy for JC-1 fluorescence. The mitochondrial membrane potential in control protoscolex cells was heterogeneous between 6 to 36 h of Met-treatment. Only following 48 h Met treatment, the energetic state of parasite cells was metabolically synchronized and the relative values of red/green JC-1 fluorescence ratios showed low dispersion. At this point, untreated protoscoleces showed a ratio of red to green fluorescence with a mean value of 3.1 (Fig 3Aa-d and 3B), whereas Met-treated protoscoleces showed a lower mean ratio of around 1.2 (Fig 3Ae-h and 3B). Metformin treatment induced an increase in depolarized regions indicated by the disappearance of red fluorescence and an increase in green fluorescence (Fig 3Ae-h).

### Occurrence and expression of genes encoding *E*. *granulosus* AMPK

Maintaining mitochondrial membrane potential is required for ATP production. By depolarizing mitochondria, Met may increase the cellular AMP:ATP ratio and modulate AMP- or ADP-sensitive enzymes such as AMPK. To investigate this possibility, we first analyzed the occurrence of the three subunits of AMPK in *E*. *granulosus* larval stages.

**Fig 4:**
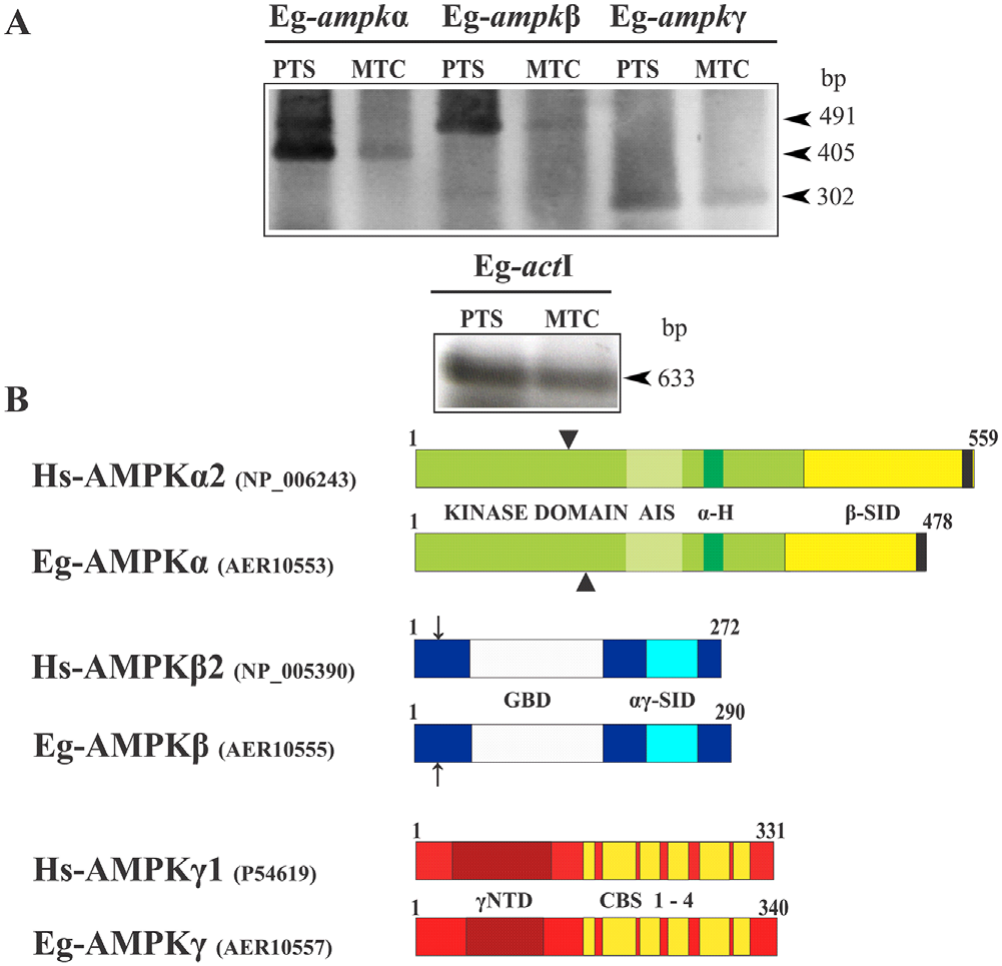
Expression and structural features of the subunits of *E*. *granulosus* AMPK. (A) Reverse transcription-PCR analysis of the three subunits of Eg-AMPK (α, β and γ) from total RNA of protoscoleces (PTS) and metacestodes (MTC). Amplification of Eg-*act*I was used as a loading control. Molecular sizes of amplicons are indicated with arrowheads. (B) Schematic representation of *Homo sapiens* AMPKα1, AMPKβ2 and AMPKγ1, and of the predicted AMPK protein from the *E*. *granulosus* genome. Identification of kinase domain, β-subunit interaction domain (β-SID), autoinhibitory sequence (AIS), α-hook (α-H) and nuclear exportation sequence (black box) in the catalytic subunit; glycogen-binding domain (GBD) and α- and β-subunits interaction domain (αγ-SID) in the β-regulatory subunit; and divergent N-terminal domain (γNTD) and cystathionine-beta-synthase repeats (CBS 1–4) in the γ-regulatory subunit.

Extensive BLASTp searches on the available *E*. *multilocularis* genome and the incompletely assembled *E*. *granulosus* genome revealed three genes coding for the different subunits of AMPK (S2 and S3 Figs). The selection of orthologs was based on reciprocal best hits in BLAST searches, using an E-value cutoff ≤1e^-25^. These coding regions were cloned, fully sequenced and annotated in GenBank (JF412830-Eg-*ampk*α-, JF412832-Eg-*ampk*β- and JF412834-Eg-*ampk*γ-). The genes encode a 478-amino acid protein (named Eg-AMPKα and annotated as AER10553), a 290-amino acid protein (named Eg-AMPKβ and annotated as AER10555) and a 340-amino acid predicted protein (named Eg-AMPKγ and annotated as AER10557). We confirmed by RT-PCR that the three genes identified were transcribed in protoscoleces and metacestodes (Fig 4A). The deduced amino acid sequences for the three subunits of the *E*. *granulosus* AMPK showed that all domains corresponding to specific functions were conserved, including key amino acids involved in protein-protein interactions (Fig 4B, S2 and S3 Figs).

The predicted Eg-AMPKα sequence aligned with 56, 61 and 97% identity with the *H*. *sapiens* (NP_006243), *Bombyx mori* (ABQ62953) and *E*. *multilocularis* (AER10552) orthologs, respectively (Fig 4B and S2A Fig). The Eg-AMPKα subunit presents a conserved N-terminal kinase domain as well as a β-subunit interaction domain (β-SID), which is found near the C-terminal end and is followed by a conserved nuclear export sequence. It also presents a highly conserved threonine residue in the activation loop of the kinase domain (Thr^176^), which is a potential phosphorylation site that could modulate the enzymatic activity. On the other hand, Eg-AMPKβ aligned with 54, 58 and 99% identity with the *H*. *sapiens* (NP_005390), *B*. *mori* (NP_001103403) and *E*. *multilocularis* (AER10554) orthologs, respectively (Fig 4B and S2B Fig). The Eg-AMPKβ subunit consists of a glycogen-binding domain, which is located in the middle of the protein and contains conserved key residues for their interaction with glycogen in both identity and position. At its C-terminal end, this protein also contains a conserved domain that presumably mediates the interaction with both the α and γ subunits of AMPK (αγ-SID). Finally, the Eg-AMPKγ subunit showed 56, 48 and 82% identity with the *H*. *sapiens* (P54619), *B*. *mori* (NP_001119720) and *E*. *multilocularis* (AER10556) orthologs respectively, and it contains the cystathionine-beta-synthase repeats that constitute the Bateman domains, with the residues involved in nucleotide binding (Fig 4B and S3 Fig).

### Pharmacological activation of Eg-AMPKα and immunolocalization in protoscoleces

**Fig 5:**
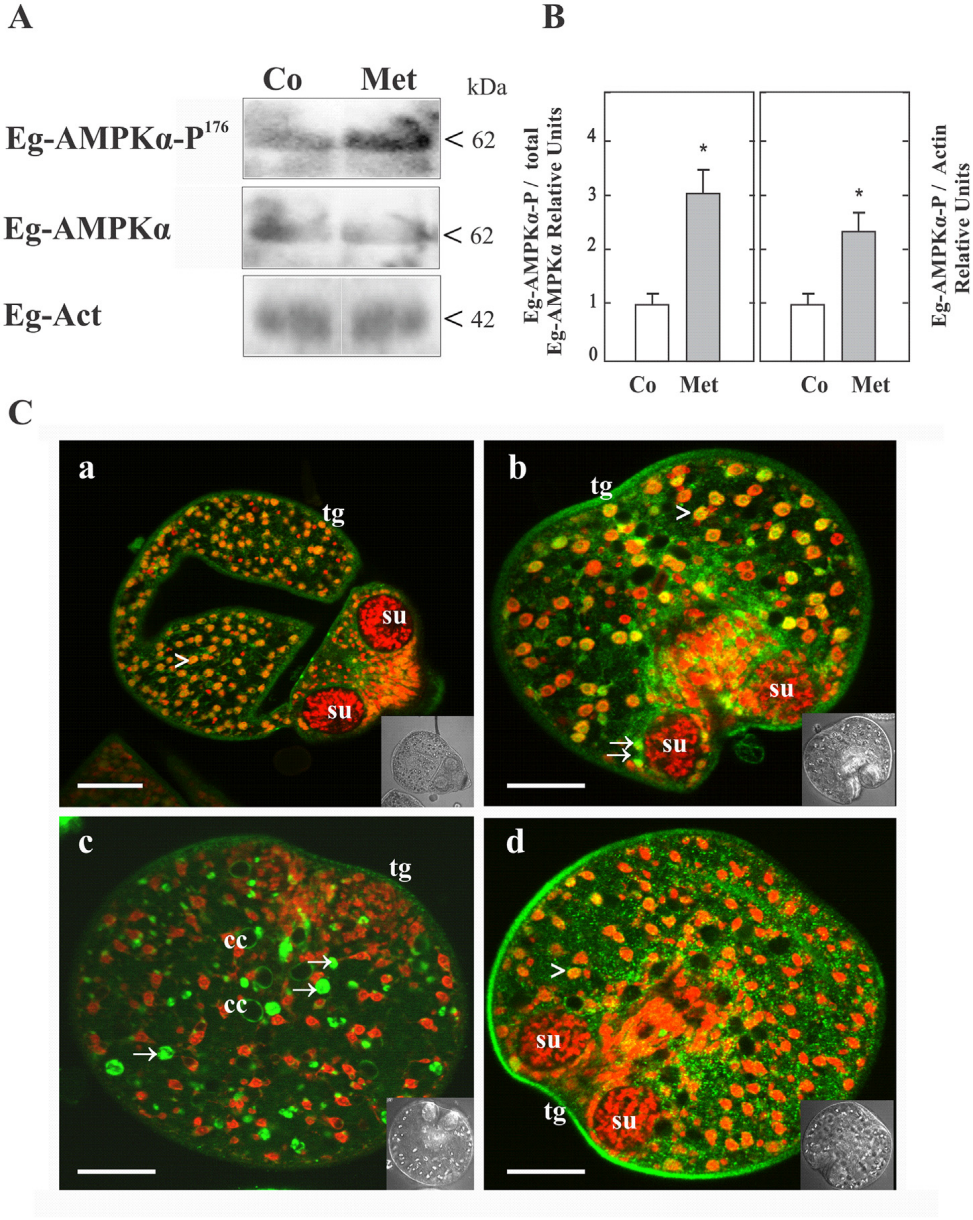
Detection and immunolocalization of total and phosphorylated Eg-AMPKα forms from *E*. *granulosus* protoscoleces. (A) Representative immunoblots of Eg-AMPKα and Eg-AMPKα-P^176^ revealed with heterologous antibodies against the total and phosphorylated forms of human AMPKα are shown. Total protein extracts from control (Co) and 10 mM metformin-treated protoscoleces (Met) were loaded at 100 μg of total protein/lane. Both Eg-AMPKα and Eg-AMPKα-P^176^ were detected (see in S2A Fig the epitopes recognized by each antibody). Polypeptide sizes are shown. (B) Graphs depict the fold change in the p-AMPK/AMPK. Densitometric analysis of Eg-AMPKα-P^176^, normalized to total AMPKα (left) and normalized to actin (right) in protoscoleces treated with 10 mM Met relative to controls (3 independent experiments with 3000 protoscoleces per sample). Values are expressed as means ± SEM (*p* < 0.05 compared to control). (C) Confocal images of *in toto* immunolocalization assays revealed with an antibody conjugated with Alexa 488—green fluorescence- and counterstained with propidium iodide—red fluorescence-. Control (a,b) and Met-treated protoscoleces (c,d) incubated with anti-AMPKα antibody (a,c) or anti-AMPKα-P antibody (b,d). Cytoplasmic expression is observed in green (arrows). Nuclear expression is observed in yellow/orange, corresponding to the merged fluorescences (arrowheads). Inset images correspond to transmission microscopy. tg: tegument; su: sucker; cc: calcareous corpuscle. Bars indicate 50 μm.

Metformin might activate Eg-AMPK as a consequence of cellular energy charge depletion. Thus, we studied the phosphorylation at Thr^176^ of Eg-AMPKα (AMPKα-P^176^) as a read-out of its activation state (Fig 5A). For that, immunoassays using rabbit monoclonal antibodies directed against the total and phosphorylated form of human AMPKα (Thr^172^) were performed from protein extract of protoscoleces and signals were normalized to total AMPKα and to actin detection (Fig 5B). We showed that a significant increase in the Eg-AMPKα-P^176^ level was observed after 48 of treatment with 10 mM Met indicating Eg-AMPKα activation under this condition. The bands were not observed when the strips were incubated with the secondary antibody alone (data not shown).

By *in toto* immunolocalization assays from protoscoleces, the expression of total and phosphorylated Eg-AMPKα forms was detected in the tegument, the posterior bladder and surrounding the calcareous corpuscles (Fig 5C). In addition, both forms were observed in the nucleus and in the cytoplasm of the cells of Met-treated and control samples (S4 Fig and data not shown), although in Met-treated protoscoleces the tegumental and nuclear Eg- AMPKα-P^176^ expression was higher than in the control condition (Fig 5Cb-d). This is consistent with the presence of a nuclear export sequence at the C-terminus of the catalytic subunit of Eg-AMPK and its direct involvement in transcriptional regulation. The fluorescence pattern was not observed when the parasites were incubated with the secondary antibody alone (data not shown).

Finally, an ortholog to human LKB1, the main kinase phosphorylating the AMPK activation loop under conditions of energy stress, was identified and shown to be constitutively expressed in *E*. *granulosus* larval stages (S5A Fig). Its predicted sequence showed 37, 37 and 94% identity with the *H*. *sapiens* (Q15831), *B*. *mori* (NP_001119722) and *E*. *multilocularis* (EmuJ_000365800) orthologs, respectively. Eg-LKB1 contains a conserved kinase domain and a nuclear localization signal at the N-terminal half of the molecule, a regulatory domain at the C-terminal and one key residue involved in autophosphorylation (T^324^, S5B–S5C Fig).

### Expression pattern of Eg-AMPKα during microcyst development

**Fig 6:**
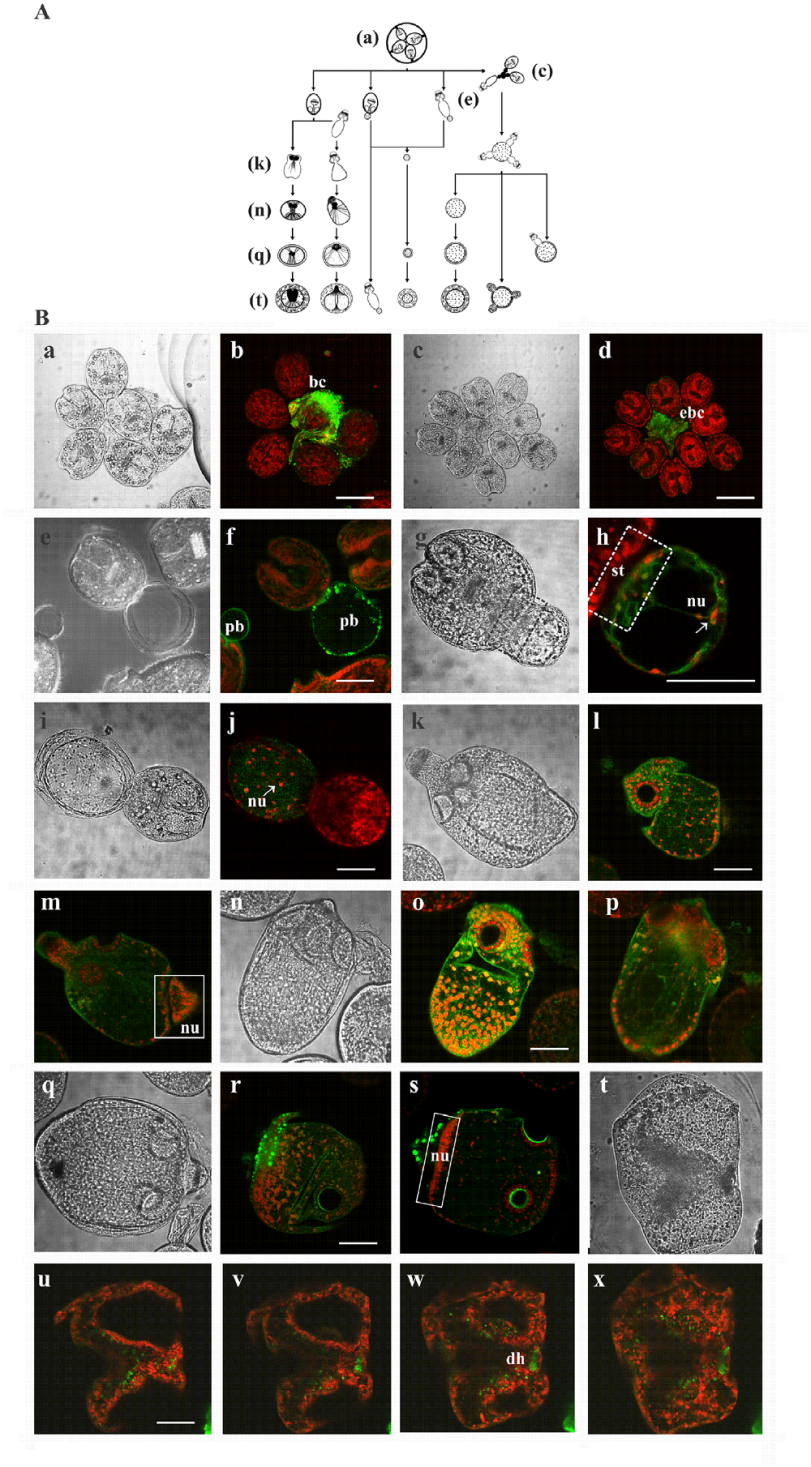
Immunolocalization of Eg-AMPKα during the *in vitro* de-differentiation process of protoscoleces to microcysts. (A) Diagrammatic representation of the different mechanisms involved in cyst development during *in vitro* culture. Image reconstructed using the figure published by Rogan and Richard (1986) [29]. This image is similar but not identical to the original image, and is therefore for illustrative purposes only. (B) Transmission and confocal microscopy of a brood capsule (a,b), an everted brood capsule (c,d), protoscoleces with posterior bladders (e,f), developing cysts from posterior bladders (g-j), vesicularized protoscoleces (k-p) and pre-microcysts developed from vesicularized protoscoleces (q-x). bc: brood capsule; ebc: everted brood capsule; pb: posterior bladder; st: stalk; nu: nucleus; dh: disrupted rostellar hook; broken-lined box indicates stalk portion; solid-lined boxes indicate distribution of nuclei in vesicularized protoscoleces. Bars indicate 200 μm (b and d) and 100 μm (f, h, j, l, o, r and u).

Under controlled *in vitro* culture conditions, protoscoleces of *E*. *granulosus* can progress in the cystic direction through different mechanisms [29] (see Fig 6A). Cysts can develop from evaginated or invaginated protoscoleces, from free posterior bladders or even from everted brood capsules. To evaluate the expression of Eg-AMPKα during the *in vitro* differentiation of protoscoleces into microcysts, we carried out *in toto* immunolocalization assays in samples collected from the same culture at different times. We were able to detect different morphological states involved in cyst development such as intact or everted brood capsules (Fig 6Ba-d), protoscoleces with posterior bladders (Fig 6Be-f), developing cysts from posterior bladder (Fig 6Bg-j), vesicularized protoscoleces (Fig 6Bk-p) and pre-microcysts developed from vesiculating protoscoleces (Fig 6Bq-x). The expression pattern of Eg-AMPKα in the structures formed during the de-differentiation process from protoscoleces was observed (Fig 6B). No signal was detected in the control samples that were only incubated with the secondary antibody under the same conditions (data not shown).

The first nuclei of the developing cyst were observed in the posterior bladder, formed from reminiscent stalk (Fig 6Bg-h). In this developmental stage, we observed high Eg-AMPKα expression on the periphery of this structure (Fig 6Be-h). Then, an increase in the number of nuclei was observed and the Eg-AMPKα expression became diffuse mainly restricted to the developing cyst (Fig 6Bi-j). On the other hand, the metamorphic events that takes place in vesicularized protoscoleces during microcyst development was also associated with changes in Eg-AMPKα expression (Fig 6Bk-s, the vesicular differentiation begins with diffuse and generalized fluorescent signal and follows with a spotted and localized expression). Finally, the pre-microcyst conserved the expression of this protein and non-specific fluorescent signal was detected in the disrupted rostellar hook (Fig 6Bt-x).

## Discussion

Chemotherapeutic attack on the energy-generating systems of parasites is a rational approach to fight parasitic infections, given that energy in the form of ATP is a commodity that these organisms cannot directly obtain from the host [30]. We reported for first time, the *in vitro* susceptibility of *E*. *granulosus* larval stages to a biguanide. In short-term assays, Met showed anti-echinococcal effects on parasites maintained in a nutrient-repleted medium, where high concentrations of the drug (starting from 1 mM in metacestodes and from 5 mM in protosocoleces, Fig 1A–1C) are required in order to affect the energy-generating systems and to interfere with the regulation of the AMPK/TOR axis, after crossing the tegumental system and to achieve the tissue distribution [31]. In this regard, doses in the millimolar range may be required to accomplish cell death effects in different human cancer cell lines [32–37]. In addition, since Met exhibits a hydrophilic nature and a slow absorption kinetic, it is considered a compound with scarce efficacy [3]. However, its combination with classic chemotherapeutic drugs allows the use of lower doses [38, 39]. Taking into account the structural and cellular differences with helminths, it has been reported that in protozoan parasites different biguanides (proguanil, chlorproguanil, synthalin, and Met) showed in the micromolar range synergistic action with atovaquone, both drug types associated with reductions in mitochondrial function, resulting in cellular damage and death [40]. In the same line of evidence, our *in vitro* pharmacological experiments showed an improved anti-echinococcal activity of Met in combination with low-dose ABZSO, compared with both drugs alone (Fig 1A and 1B). Likewise, it was necessary to use at least 1 mM Met to achieve the *in vitro* therapeutic effect. Therefore, the true biological effects of this drug can be resolved with further *in vivo* assays, including also combinations of Met with conventional anti-echinococcal agents such as benzimidazoles.

Since Met is actively transported into cells by the organic cation transporters, called OCT1/OCTN1 or SLC22A1/4 [41, 42], the accumulation of the drug is significantly higher in tissues, particularly in gut and liver (~ 200 μmol/kg wet weight of tissue) than in plasma (~ 30–50 μM) [43], achieving micromolar levels in hepatocytes of the periportal zone [33, 43, 44]. In this work, we identified the putative OCTs (EgrG_001058900 and EgrG_000957000) in the *E*. *granulosus* genome, which displayed structural similarities and 25% identity with the *H*. *sapiens* ortholog. Given that the liver is the main tissue of action of Met and a target organ in hydatidosis, it will be interesting to carry out effectiveness studies of Met with *in vivo* models using a Met concentration that can be safely obtained in the clinical setting (50 mg/kg day) [43], which will give us better insights into the action of this drug in the cestode.

In human cell culture and in xenograft models, Met can interfere with cell cycle progression, leading to G0/G1 or S phase arrest, through a decrease in cyclin D1 protein levels [32]. However, the most accepted anti-proliferative effect of Met is via regulation of the AMPK/TOR axis under control of LKB1 [45–47]. In consequence, the suppression of TORC1 signaling by AMPK agonists has therapeutic implications for the treatment of human cancer using existing FDA-approved agents [48]. Since we have previously reported conserved TORC1 in *Echinococcus* [20, 21], in this work Met was used as the first criterion to identify AMPK-controlled events in *Echinococcus* larval stages. Based on the drug susceptibility and using information obtained from the *Echinococc*us genome project, one gene encoding for the catalytic subunit (Eg-*ampk*) and for each regulatory subunit (Eg-*ampk* and Eg-*ampk*) were identified and their constitutive expression verified, which allowed us to clone their transcripts (Fig 4, S2 and S3 Figs). Additionally, in accordance to what was found by Zheng et al. [49], our results showed that the genes encoding Eg-AMPK subunits have a higher expression level in protoscoleces when compared to metacestodes (Fig 4A). Their predicted proteins, as well as the putative Eg-LKB1 protein, show considerable evolutionary conservation of the sites interacting with each other, according to their mammalian counterparts (S2, S3 and S5 Figs). Besides the *Echinococcus ampk* genes cited in this work, an additional gene encoding each regulatory subunit has been reported by Tsai et al. [25]. Regarding the *ampk* gene number, one catalytic subunit, three β-subunits and one γ-subunit have been identified in the yeast genome. On the other hand, *Caenorhabditis elegans* and *H*. *sapiens* have two α-, two β-, and five and three γ- gene orthologs, respectively, while in *Drosophila melanogaster*, each subunit is encoded by a single gene. Since AMPK functions normally and is stable as a heterotrimeric complex, the number of genes in each organism determines how many complexes can possibly be formed in each of them [13].

It has been demonstrated that AMPK is indirectly activated by Met as a consequence of respiratory chain complex I inhibition [9]. Indeed, the subcellular target of Met is the mitochondrion, in which the membrane potential might drive the accumulation of the positively charged drug within the organelle matrix. Following treatment with Met in the presence of JC-1 dye, the number of mitochondria that exhibited a low red/green ratio was markedly increased in *Echinococcus* protoscoleces (Fig 3). These findings indicate that Met causes mitochondrial membrane depolarization and could inhibit the complex I of the electron transport chain, in accordance with previous reports [9, 40, 50]. In fact, it has been previously reported that impairment of mitochondrial respiration by complex I blockade with nafuredin and quinazoline-type compounds in *Ascaris suum* and *E*. *multilocularis* also shows anti-parasitic effects [51–53].

In *E*. *granulosus* Met-treated protoscoleces, a decreased mitochondrial ATP production might induce an increase in larval AMP levels and subsequent Eg-AMPK activation through phosphorylation on T^176^ (Fig 5A and 5B), potentially via Eg-LKB1. The ATP:AMP ratio of the *Echinococcus* protoscoleces has been reported to be in the range of 0.86–0.92, the highest value recorded for a parasite [54]. Nevertheless, in our study, the high Eg-AMPK activity in the protoscolex basal state might reflect an increased AMP content, even under nutrient-rich conditions. On the other hand, a decrease in oxidative phosphorylation is equivalent to nutrient depletion in terms of ATP supply and could force the cells to engage survival processes such as increased glycolysis and autophagy [55]. Since AMPK promotes autophagy from yeast to mammals [56–58], further experiments with hydatid fluid and different starvation conditions should be carried out to study AMPK/TOR signal pathways in this cestode.

As a consequence of reduced ATP production, glycolysis and glycogenolysis are stimulated. Indeed, both catabolic processes increase in Met-treated mammalian cells [59]. In this line of evidence, we showed that Met induces a decrease in glycogen levels, the major energy reserve in parasite tissue, and an increase in Eg-α-amylase and Eg-LDH activities, indicating an increase in these catabolic processes in *Echinococcus* larval stages (Fig 2A and 2B). On the other hand, our results also showed a decreased expression of Eg-*pepck*, which encodes a CO_2_-fixing enzyme that participates in the glycolytic pathway of parasitic helminthes [30, 60, 61], and Eg-MDHc, a critical enzyme in the malate—aspartate conversion, both key enzymes in the intermediary metabolism of cestodes [30, 62]. This could be affecting the generation of cytosolic and mitochondrial NADH/NAD^+^, further compromising the ATP pools in *Echinococcus* Met-treated cells and causing metabolic exhaustion in the parasite. Also, we demonstrated that Eg-*g6p* and Eg-*f1*,*6bp* are down-expressed in drug-treated protoscoleces and metacestodes (Fig 2C and 2D). In this regard, the transcription of genes encoding two key hepatic gluconeogenic enzymes, PEPCK and G6P is inhibited in rat hepatocytes by Met-dependent activation of AMPK [59, 63, 64]. Given that, in parasitic helminthes the physiological role of PEPCK is the carboxylation of phosphoenolpyruvate to form oxaloacetate, it is not comparable to its function in mammals, where the enzyme catalyzes the inverse reaction during the gluconeogenesis [30, 65]. Although these parasites rely on stored glycogen for energy, the occurrence of gluconeogenesis has not been demonstrated in *Echinococcus* larval stages yet, nor has the transcription of carbon-metabolism related genes been studied.

In particular, the simultaneous action of glycogenolysis, homolactic fermentation and malate dismutation are linked to the Warburg effect in *Echinococcus* cyst germinal cells, facilitating the uptake and incorporation of nutrients into the biomass during cell division [65]. Indeed, the germinal layer converts most glucose to lactate through aerobic glycolysis, regardless of whether oxygen is present. Notably, AMPK has been shown to negatively regulate the Warburg effect *in vivo*, the change from oxidative metabolism to glycolysis frequently detected in tumor cells [6, 66]. This could be another cause of the pharmacological effect of Met on *E*. *granulosus* metacestodes (Fig 1B). Since Eg-AMPKα expression was detected during the differentiation of protoscoleces towards microcysts (Fig 6B), this drug also could represent an anti-echinococcal alternative during the development of secondary hydatidosis.

Finally, the subcellular expression of AMPK has important functional consequences depending on the substrate location. In coordination with AMPK-dependent events, LKB1 kinase also shuttles in and out of the nucleus [16]. Here, we demonstrated by *in toto* immunoassays that total and phosphorylated Eg-AMPKα was expressed both in the nucleus and cytoplasm of the cells (Fig 5C and S4 Fig). This is consistent with the presence of a nuclear export sequence in both Eg-AMPKα and Eg-LKB1 (S2A and S5B Figs). Thus, AMPK-downstream actions could exert a dual control over cellular metabolism and transcriptional regulation [13]. At nuclear level, in mammalian cells and worms, AMPK affects the transcription by phosphorylation of various transcription factors, such as FoxO family proteins [15, 67]. Recently, we identified a single FoxO transcription factor in the *E*. *granulosus* genome [21] verifying that its amino acid sequence presents the six potential conserved phosphorylation sites for Eg-AMPK (T^278^, S^509^, S^522^, S^647^, S^680^, S^714^), as it happens in mammals [67, 68].

Given its sensitivity to the energy state of the cell and the whole organism, AMPK could be highly relevant to parasitic helminths, which adjust their metabolism to a low rate of ATP turnover [69, 70]. Thus, the understanding of the nature of different carbohydrate-energy regulatory mechanisms in these parasites represents a solid basis for choosing appropriate targets for new chemotherapeutic agents.

## Supporting Information

S1 Table Primers used to amplify encoding genes for LKB1, AMPK, G6P, F1,6BP, PEPCK and MDHc in *E*. *granulosus*. (DOC)

S1 Fig Multiple sequence alignment of α-amylase proteins including *E*. *granulosus*. Consensus is indicated in the last line, total (uppercase letter), conservative changes (numeral) and absence of consensus (dots) and gaps introduced to maximize the alignment (dashes). Characteristic catalytic residues Asp^229^, Glu^258^ and Asp^323^ are indicated with arrowheads and six of the seven characteristic motifs of the conserved regions of Taka-amylase A are indicated with boxes [27]. GenBank accession numbers for the α-amylase proteins are: Taka-amylase-A, *Aspergillus oryzae* (0901305A) and Eg-α-amylase *E*. *granulosus* (AEJ15816).
(TIF)

S2 Fig Multiple sequence alignment of AMPKα (A) and AMPKβ (B) orthologs including *Echinococcus* spp. Consensus is indicated in the last line, total (uppercase letter), partial (lowercase letter), conservative changes (asterisk), absence of consensus (dots) and gaps introduced to maximize the alignment (dashes). (A) Catalytic subunit of Eg-AMPK shows a characteristic phosphorylation site (Thr^176^ black arrowhead) surrounded by a highly conserved region (gray box, coincident with the amino acids that recognizes the Phospho-AMPKα antibody utilized in the immunoassays), a segment called α-hook (link between the kinase domain and the C-terminal regulatory fragment) that might interact with the γ-subunit (double dotted-underlined, 349–355) [71], an autoinhibitory sequence (solid-underlined, 298–335) which might bind to the kinase domain through of conserved residues in both regions (L^74^, R^76^, Y^133^, R^136^, R^266^ indicated by numeral and V^299^, L^314^, L^323^, L^329^, D^332^, N^333^ indicated by arrows), [72] and a C-terminal nuclear export sequence (NES) (broken-lined box, 465–478 with key leucine-L^472^, L^476^- indicated by arrows) [16]. In addition, in the alignment is indicated with a solid box, the amino acid region (surrounding Lys^40^-white arrowhead- of human ortholog) that recognizes the total AMPKα antibody utilized in the immunoassays. GenBank accession numbers for the AMPKα proteins are: Bm, *Bombyx mori* (ABQ62953), Hs, *Homo sapiens* (NP_006243), Eg, *Echinococcus granulosus* (AER10553) and Em, *Echinococcus multilocularis* (AER10552). (B) Eg-AMPKβ consists of a glycogen binding domain (underlined, 118–173) with conserved key residues (W^118^, S^129^, K^147^, W^154^, N^172^, indicated by arrows) [73], an N-terminal consensus sequence for myristoylation (MGNXXS/T, gray box) associated with facilitating membrane binding [74] and a conserved H^253^ (arrowhed). GenBank accession numbers for the AMPKβ proteins are: Bm, *Bombyx mori* (NP_001103403), Hs, *Homo sapiens* (NP_005390), Eg, *Echinococcus granulosus* (AER10555) and Em, *Echinococcus multilocularis* (AER10554).
(TIF)

S3 Fig Amino acid sequence comparison between *Echinococcus* AMPKγ and metazoan orthologs. Consensus is indicated in the last line, total (uppercase letter), partial (lowercase letter), conservative changes (asterisk), absence of consensus (dots) and gaps introduced to maximize the alignment (dashes). Eg-AMPKγ presents four cystathionine β-synthase (CBS) motifs: CBS1 (underlined, 75–154), CBS2 (gray boxes, 53–74 and 155–209), CBS3 (broken-lined box, 230–300) and CBS4 (solid-lined boxes, 210–229 and 301–355) [75]. This protein conserves key residues involved in binding to adenine nucleotides (R^96^, D^116^, H^176^, R^177^, K^195^, R^196^, T^225^, S^251^, D^270^, H^323^, R^324^, S^344^, indicated by arrows) [76] and a pseudosubstrate sequence within the CBS2 sequence (L ^164^DAV ^167^QMLL ^171^EHKV ^175^HR ^177^LPILDPE, delimited by arrowheads) [73]. GenBank accession numbers for the AMPKγ proteins are: Bm, Bombyx mori (NP_001119720), Hs, *Homo sapiens* (P54619), Eg, *Echinococcus granulosus* (CDJ18193) and Em, *Echinococcus multilocularis* (AER10556).
(TIF)

S4 Fig Subcellular immunolocalization of total Eg-AMPKα from control *E*. *granulosus* protoscoleces. Images of protoscoleces (i-l) and soma (a-d and m-p) and scolex (q-x) regions visualized by fluorescence confocal microscopy stained with propidium iodide—red fluorescence, first column on the left-, revealed with AMPKα antibody conjugated with Alexa 488-green fluorescence, second column-, obtained by overlapping of the two fluorescence reactions (third column) and visualized by light transmitted microscopy (last column on the right). The punctuate staining for Eg-AMPK-α expression was evenly detected in both nucleus (a-k, arrowheads) and cytoplasm (m-x, asterisk). Nuclear expression is observed in yellow/orange, corresponding to the merged fluorescences (g and k, arrows). Bars indicate 5 μm (j-r), 10 μm (a-c) and 50 μm (d-i), tg: tegument; su: sucker; bo: cell body; rc: rostellar cone; cc: calcareous corpuscle.
(TIF)

S5 Fig Structural organization, sequence alignment and expression of LKB1. (A) Reverse Transcription-PCR assay of Eg-*lkb1* gene from total RNA of protoscoleces (PTS) and metacestodes (MTC). Molecular size of amplicon is indicated with arrowhead. (B) Schematic representation of *Homo sapiens* LKB1 and of the only predicted LKB1 protein from the *E*. *granulosus* genome. Identification of N-terminal regulatory domain (blue), kinase domain (red) with activation loop (LAc, green), proline-rich C-terminal flanking tail (CFT_L_, orange) and C-terminal regulatory domain (CDR, yellow). The proteins show conserved nuclear localization signal (indicated by a cross) and key residues involved in autophosphorylation (indicated by arrows). (C) Multiple alignment of LKB1 orthologs. Consensus is indicated in the last line, total (uppercase letter), partial (lowercase letter), conservative changes (asterisk), absence of consensus (dots) and gaps introduced to maximize the alignment (dashes). Eg-LKB1 presents a kinase domain (underlined, 94–526) with conserved residues in the LAc (A^384^, D^387^, T^409^, P^497^, P^498^, P^499^, indicated by numeral) and key residues for catalysis and substrate binding (D^373^, N^320^, D^315^, T^395^, H^313^, indicated by arrowheads), a CFT_L_ (gray box, 491–527) and a CDR (broken-lined box, 530–622). Sequence also contains residues involved in the structural integrity (R^126^, K^130^, R^138^, E^150^, D^176^, L^303^, I^316^, L^321^, A^381^, F^382^, L^425^, Y^452^, L^465^, W^512^, indicated by arrows) [77]. GenBank accession numbers for the LKB1 proteins are: Bm, *Bombyx mori* (NP_001119722) and Hs, *Homo sapiens* (Q15831). GeneDB predicted protein: Eg, *Echinococcus granulosus* (EgrG_000365800) and Em, *Echinococcus multilocularis* (EmuJ_000365800).
(TIF)
