# Supplementary material for: In Vitro Anti-Echinococcal and Metabolic Effects of Metformin Involve Activation of AMP-Activated Protein Kinase in Larval Stages of Echinococcus granulosus
Source: PLoS One. 2015 May 12;10(5):e0126009. doi: 10.1371/journal.pone.0126009 (PMC4429119; doi:10.1371/journal.pone.0126009)
Supplement: S1 Table — (DOC) [file pone.0126009.s001.doc]

**S1 Table. Primers used to amplify encoding genes for LKB1, AMPK, G6P, F1,6BP, PEPCK, MDHc, α-amylase and actin I in *Echinococcus granulosus*.**

| Gene | Primer | Sequence 5´- 3´ | Product size (bp) |
| --- | --- | --- | --- |
|  |  |  |  |
| *Eg-lkb1* | *Eg-lkb1-Fw* | 5´- GATCGTCTCTAGACTTGGGGTTCGAAAAATTC -3´ | 584 |
| *Eg-lkb1* | *Eg-lkb1-Rv* | 5´- GTGTGATTAGCAGATTGGCCGGTTTGATATC -3´ |
|  |  |  |  |
| *Eg-ampkα* | *Eg-ampkα-Fw* | 5´- GACGTTTGGTCTTGTGGTGTGATTCTTTAC -3´ | 405 |
| *Eg-ampkα* | *Eg-ampkα-Rv* | 5´- CTTGTTGTCAATGATCAGATGATAGGCGAC -3´ |
|  |  |  |  |
| *Eg-ampkβ* | *Eg-ampkβ-Fw* | 5´- CCAATGGTGGATAACGAGTATGGGACAAGG -3´ | 491 |
| *Eg-ampkβ* | *Eg-ampkβ-Rv* | 5´- GTTGTCTATGGGCTTGTAGAAAAGAGTGGAG -3´ |
|  |  |  |  |
| *Eg-ampkγ* | *Eg-ampkγ-Fw* | 5´- GCTTTTTGGCTCTCGTTTATAATGGCGTTAG -3´ | 302 |
| *Eg-ampkγ* | *Eg-ampkγ-Rv* | 5´- CAAAATGGGAAGACGGTGTACCTTGTGTTC -3´ |
|  |  |  |  |
| *Eg-g6p* | *Eg-g6p-Fw* | 5´- ATGGAGTGTATCTACCTTTATGGTGCTAAG -3´ | 555 |
| *Eg-g6p* | *Eg-g6p-Rv* | 5´- CAGTGTGTACCGCATCGTCCATTGATAGAAG -3´ |
|  |  |  |  |
| *Eg-f1,6b* | *Eg-f1,6bp-Fw* | 5´- GAAGAAGTGAAGAAGCTAGATGTCATTTCC -3´ | 445 |
| *Eg-f1,6b* | *Eg-f1,6bp-Rv* | 5´- CTTCGTTAATGGAGTAAATTTTGCCGCGTTTG -3´ |
|  |  |  |  |
| *Eg-pepck* | *Eg-pepck -Fw* | 5´- CCGAAGCCGATAAGATCACTGACGAACTTC -3´ | 800 |
| *Eg-pepck* | *Eg-pepck-Rv* | 5´- CACGCAATGTCATCACCAACACACATAACC -3´ |
|  |  |  |  |
| *Eg-mdh_c_* | *Eg-mdh_c_-Fw* | 5´- ATCTGTTCGGCAAAGACCAGCAGATCATTCTCCACCTC -3´ | 776 |
| *Eg-mdh_c_* | *Eg-mdh_c_-Rv* | 5´- GAAGTAGATGTCCTTGGGGGCACCATAGTGAT -3´ |
|  |  |  |  |
| *Eg-amylase* | *Eg-amyl-Fw* | 5´- CAATCGAAAGTTGGCTACTGGGTAAATC-3´ | 243 |
| *Eg-amylase* | *Eg-amyl-Rv* | 5´- CAACAATGCACCCAAATCATCCATG -3´ |
|  |  |  |  |
| *Eg-actin I* | *Eg-act*I-*Fw* | 5´-GCGATGTATGTAGCTATCCAGGCAGTGCTCTCGCT-3´ | 633 |
| *Eg-actin I* | *Eg-act*I-*Rv* | 5´-CAATCCAGACAGAGTATTTGCGTTCCGGAGGA-3´ |
